# Association between *PAX9* or *MSX1* gene polymorphism and tooth agenesis risk: A meta-analysis

**DOI:** 10.1515/biol-2022-0987

**Published:** 2025-04-10

**Authors:** Xiaoyi Zhong, Kaixin Liu, Zhenmin Liu, Cuiping Li, Wenxia Chen

**Affiliations:** Hospital of Stomatology, Guangxi Medical University, Nanning, 530021, Guangxi, China; Department of Endodontics, College & Hospital of Stomatology, Guangxi Medical University, Nanning, 530021, China; Department of Conservative Dentistry & Endodontics, College of Stomatology, Guangxi Medical University, Nanning, 530021, Guangxi, China; Guangxi Key Laboratory of Oral and Maxillofacial Rehabilitation and Reconstruction, Nanning, 530021, Guangxi, China; Guangxi Health Commission Key Laboratory of Prevention and Treatment for Oral Infectious Diseases, Nanning, 530021, Guangxi, China

**Keywords:** tooth agenesis, gene polymorphism, *PAX9*, *MSX1*, meta-analysis

## Abstract

Tooth loss represents the most prevalent form of dental agenesis. Anterior tooth loss primarily impacts aesthetics and psychological well-being, whereas posterior tooth loss influences bone growth patterns and masticatory function. Prolonged tooth loss can significantly hinder subsequent restorative procedures. Genetic factors are among the principal contributors to tooth loss, as genes dictate the location, quantity, and morphology of teeth; mutations at specific gene loci may result in underdevelopment or even complete absence of teeth. Investigating the relationship between gene polymorphisms and tooth loss could yield novel insights for future clinical interventions aimed at addressing this issue. Consequently, this study aims to elucidate the correlation between *PAX9* and *MSX1* gene polymorphisms and instances of tooth loss. We searched Cochrane, PubMed, Web of Science, MEDLINE, EMBASES, and CNKI journal databases for articles up to April 1, 2024 to determine the association of *PAX9* and *MSX1* genes with the risk of dental development. Used STATA version 11.2 to calculate the odds ratio (OR) and 95% confidence interval (CI). Analyzed meta-regression, sensitivity, and publication bias. Used Bayesian measures of the false positive reporting probability and false discovery probability to examine the reliability of the calculation. Finally, 12 eligible reports were included in this study, including 7 reports on *PAX9 rs2073247*, with 873 cases of polymorphism and 812 cases of control; 5 reports on *PAX9 rs2073244*, with 668 cases of polymorphism and 668 cases of control; 7 reports on *MSX1 rs12532*, with 762 cases of polymorphism and 1,544 cases of control. The ORs and 95% CIs showed a statistically significant relationship between *PAX9 rs2073247* or *PAX9 rs2073244* polymorphism and tooth agenesis risk. Moreover, there was no association observed for the *MSX1 rs12532* polymorphism. In further subgroup analysis of the polymorphisms (*PAX9 rs2073247*, *PAX9 rs2073244*), we found an increased risk of tooth loss in the Caucasian and Hungarian groups. This article concludes that the *PAX9 rs2073247* and *PAX9 rs2073244* polymorphism might help to increase the risk of tooth agenesis. Understanding the mechanisms of genetic mutations will enable clinical physicians and human geneticists to develop new strategies for future therapeutic research and preventive treatments.

## Graphical abstract

**Figure:**
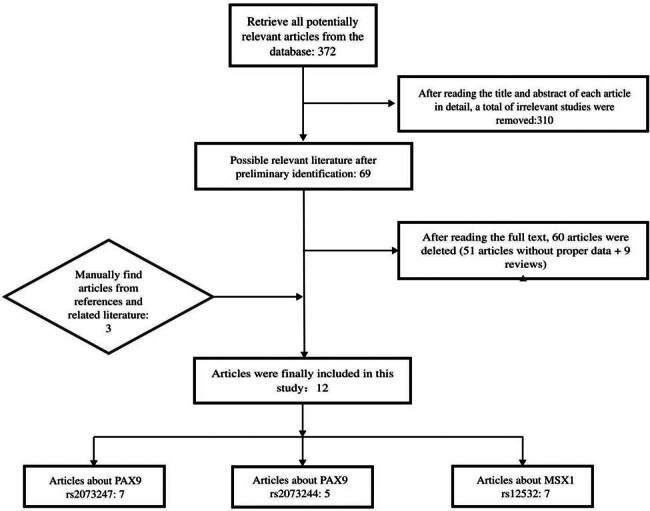

## 1 Introduction

Tooth loss is the most common type of dental malformation currently. When fewer than six teeth are missing (excluding the third permanent molar), it is diagnosed as hypodontia. Oligodontia and agenesis are usually referred to as the loss of more than six teeth (excluding the third permanent molar) [1,2]. The absence of front teeth primarily affects aesthetics and mental health, while the loss of rear teeth impacts bone growth patterns and chewing function. Long-term loss of teeth even brings great inconvenience to restoration. As early as six decades ago, scholars pointed out that tooth loss was related to genetics, and more than 300 genes were involved in the development of teeth, including *MSX1*, *PAX9*, *AXIN2*, *EDA*, *SPRY2*, *TGFA*, *SPRY4*, *WNT10A*, *FGF3*, *FGF10*, *FGFR2*, and *BMP4*. These genetic systems determine the position, number, and shape of teeth. Notably, *MSX1* and *PAX9* exhibit higher mutation frequencies. The simplest and most common form of genome-level DNA variation between individuals is the substitution of one nucleotide for another, known as a single nucleotide polymorphism (SNP) [3]. The probability of allelic variation or mutation in individuals is about 0.1%. Paired box transcription factors (*PAX*) and their homologs, ubiquitous in vertebrates and invertebrates, play a key role in various stages of embryonic development. *MSX1* is the first gene identified to be associated with human edentulous, and its regulatory role is critical in inducing nonsyndromic edentulous by interfering with DNA binding. It is known that *PAX9* and *MSX1* genes are not only related to the tooth development of the posterior teeth [4–10], but also play an indispensable role in craniofacial development and differentiation [11–14]. At the same time, studies of mouse tooth development have also shown a molecular relationship between *PAX9* and *MSX1*, which are co-expressed in the tooth mesenchyme and appear to be critical for mouse tooth morphogenesis. Ogawa et al. [15] observed that in mice with simple co-mutations of *MSX1* and *PAX9*, the expression of bone morphogenetic proteins (*BMP4*) was significantly reduced in the periodontal mesenchyme, and they also observed that in mice with homozygous ineffective mutants of *MSX1* and *PAX9*, tooth development stalled at the bud stage. As an effector molecule, *BMP4* is known to be involved in downstream signaling events that induce enamel junctions [16]. The enamel junction is a transient signaling center located in the epithelial depression of the central part of the tooth embryo during the cap stage. It controls the shape, height, position, and number of tooth cusps. Therefore, the enamel junction is called the regulatory center of tooth embryo morphogenesis and regulates the occurrence of tooth embryo morphogenesis. *MSX1* and *PAX9* are among the most extensively studied dental mesenchymal transcription factors, with over 50 mutations linked to various types of dental hypoplasia and other inherited dental defects or variations [17]. The SNPs that have been found to be most relevant to the expression level of *BMP4* are the single thymine nucleotide insertion/deletion variants in *PAX9* (T > C) and the single guanosine nucleotide insertion/deletion variants in *MSX1* (G > A). These variants may play key regulatory roles in the early stages of tooth formation [18]. Mutations in *PAX9* and *MSX1* may lead to defects in protein–protein interactions that disrupt the expression of *BMP4*, a normal downstream function essential for tooth morphogenesis, resulting in defects in tooth development. Numerous case–control studies have reported the correlation between *PAX9 rs2073247*, *PAX9 rs2073244*, and *MSX1 rs12532* gene polymorphisms and tooth agenesis. Therefore, we conducted this meta-analysis to further explore the association between these gene polymorphisms and tooth agenesis.

## 2 Materials and methods

### 2.1 Strategy for retrieving qualified articles in the database

The relationship between *PAX9 rs2073247*, *PAX9 rs2073244* and *MSX1 rs12532* gene polymorphisms and susceptibility to tooth agenesis was studied using Cochrane, PubMed, MEDLINE, WEB OF SCIENCE, and CNKI. The search terms and keywords were (1) “*PAX9*”/“*MSX1* “(Mesh), (2) “SNPs” or “SNP” or “polymorphism” or “genetic” (Mesh), (3) “hypodontia” or “oligodontia” or “tooth agenesis” (Mesh). The search will end on April 1, 2024. In order to prevent the omission of relevant important articles, we conducted a careful manual search of all eligible articles, review articles, and other relevant studies and repeated them several times. Unpublished articles will not be discussed.

### 2.2 Selection criteria of eligible articles and quality assessment

Inclusion criteria included: (1) the association of at least one of the three gene polymorphisms (PAX9 rs2073247, PAX9 rs2073244, MSX1 rs12532) with the risk of tooth agenesis was evaluated; (2) the study must be a case–control study; (3) all patients should have been diagnosed with dental dysplasia through panoramic radiographs and thorough clinical examination; (4) alleles or gene frequencies for both case and control group must be extracted; and (5) published in English or Chinese.

Exclusion criteria included: (1) insufficient data to calculate odds ratio (OR) and 95% confidence interval (95% Cl) and (2) meta-analyses, reviews, letters, and editorials.

### 2.3 Data extraction

Two independent researchers (Xiaoyi Zhong) carefully selected relevant studies according to the inclusion criteria. Another researcher (Cuiping Li) examined the extraction of raw data. For those where there is still any debate, all the studies are discussed and consensus is reached. Data collected from each article included the first author’s last name, when the paper was published, race, country, source of control, and number of genotypes. Ethnic groups are often classified as Caucasian, Asian, Hungarian, and Black.

### 2.4 Data calculation

The correlations of PAX9 rs2073244, MSX1 rs12532, and PAX9 rs2073247 gene polymorphisms with the tooth loss risk were estimated using 95% CI and OR. The combined OR was evaluated using four genetic models, including homozygote comparison (GG/AA; TT/CC), heterozygote comparison (AG/AA; CT/CC), dominant (AG + GG/AA; CT + TT/CC), and recessive (GG/AA + AG; TT/CT + CC) models in PAX9 rs2073244, MSX1 rs12532, and PAX9 rs2073247. The *I* ^2^ and *p*-values were used to calculate and analyze the heterogeneity. Heterogeneity was calculated and analyzed by using *I* ^2^ statistics and *p* value. The random effects model was used when there were statistical differences found in heterogeneity (*p* < 0.05, *I* ^2^ > 50%), or else the fixed effects model was conducted (*p* > 0.05, *I* ^2^ < 50%).

The probability of false positive reports (FPRP) is calculated to evaluate meaningful findings. We set the threshold for FPRP at 0.2% and specified prior probabilities of 0.25, 0.1, 0.01, 0.001, and 0.0001 to detect an OR of 1.5 associated with cancer risk in the study. Only if the FPRP value is less than 0.2 is the result considered noteworthy [19]. A spreadsheet was calculated using Excel to estimate the probability of Bayesian Error Finding (BFDP) to assess the confidence of a statistically significant association. A BFDP below 0.8 is considered a noteworthy [20].

## 3 Results

### 3.1 Study characteristics

**Figure 1:**
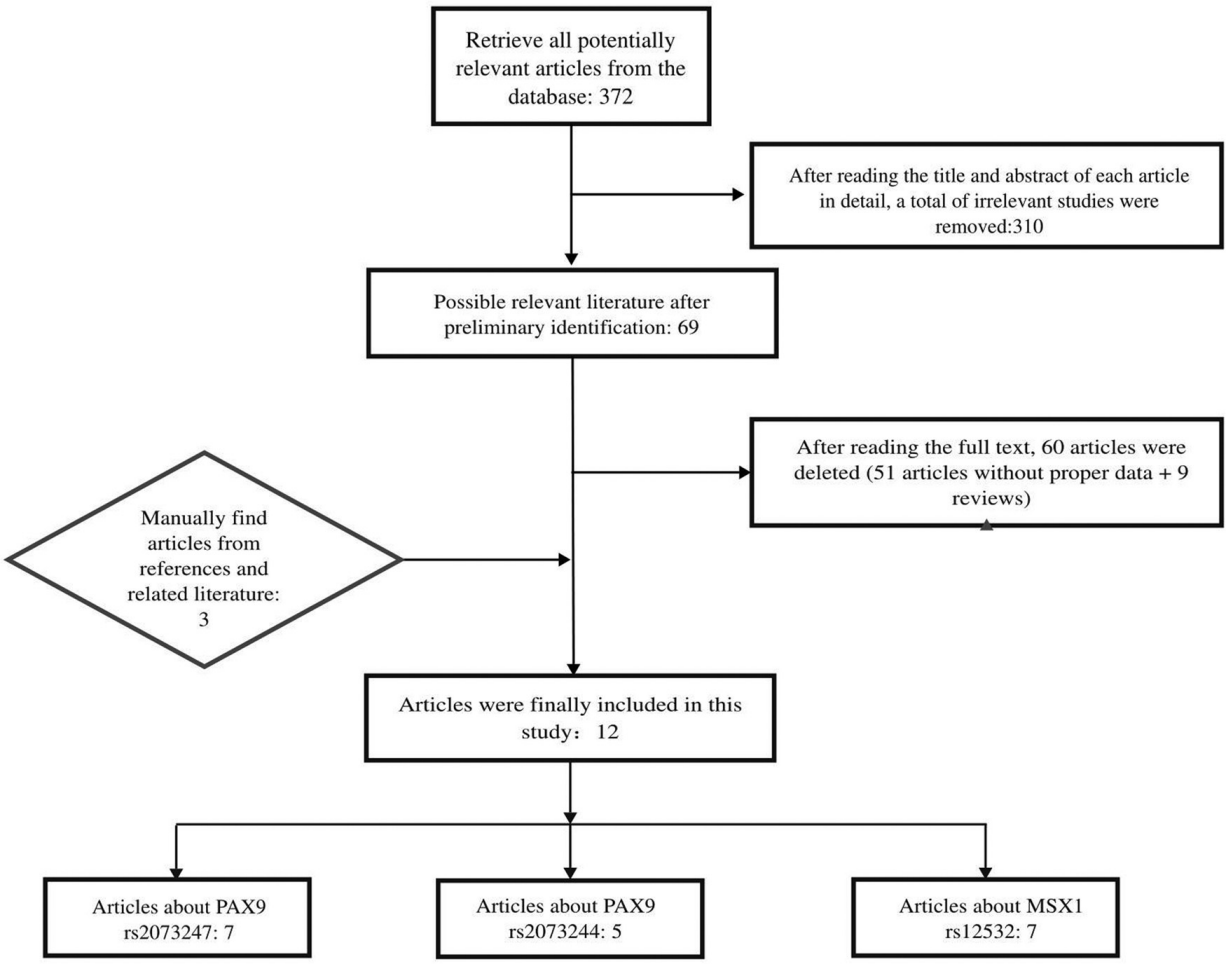
A flow chart of the entire selection document.

According to the search criteria (Figure 1), a total of 372 potentially relevant articles were screened out after eliminating duplicates through online database search. After reading the title and abstract of each article in detail, a total of 310 unrelated studies were removed. A total of 69 potential reports were collated against the reference criteria mentioned above. After reading the full text, 60 articles were deleted (51 articles without proper data + 9 reviews). Three articles were manually found from references and related literature. A total of 12 articles were retrieved, and the flow chart of the whole selection process is shown in Figure 1. There were seven studies of *PAX9 rs2073247* (873 cases and 812 control samples) [4,21–26]. Five studies on *PAX9 rs2073244* were conducted (668 cases and 668 control samples) [21,23–26]. Seven studies on *MSX1 rs12532* were found (762 cases and 1,544 control samples) [4,23,27–31].

For *PAX9 rs2073247* polymorphisms, four hospital-based studies and three population-based studies were among the seven case–control studies. There were three studies of Caucasians, two studies of Asians, and one study of Hungarian ethnicity. For *PAX9 rs2073244* polymorphism, two of the five case–control studies were hospital-based and three were population-based. In terms of ethnicity, there were three studies of Caucasians, one study of Asians, and one study of Hungarians. Regarding *MSX1 rs12532* polymorphism, seven of the seven case–control studies were hospital-based. In terms of ethnicity there were three studies on Caucasians, two on Asians, one on Hungarians, and one on Blacks. The main characteristics of *PAX9 rs2073244, PAX9 rs2073247*, and *MSX1 rs12532* polymorphisms in the included studies are shown in Table 1.

**Table 1:** Main characteristics of the eligible studies for *PAX9 rs2073244*, *PAX9 rs2073247*, and *MSX1 rs12532* polymorphisms

No.	Author	Year	Ethnicity	Country	Disease type	Source of control	Sample size of case	Sample size of control	CaAA	CaAG	CaGG	ConAA	ConAG	ConGG	MAF	Genotyping method
***PAX9 rs2073244***																
1	Ahmed	2018	1	Jordan	Non-syndromic hypodontia	2	72	72	27	35	10	43	28	1	0.36	PCR
2	Gabriella	2013	3	Hungary	Non-syndromic hypodontia	1	192	260	54	106	32	98	121	41	0.44	PCR
3	Isman	2013	1	Turkey	Non-syndromic hypodontia	2	200	114	15	109	76	12	57	45	0.4	PCR
4	Peres	2005	1	Brazil	Non-syndromic hypodontia	2	102	106	36	56	10	56	47	3	0.37	PCR
5	Yong chu Pan	2008	2	China	Non-syndromic hypodontia	1	102	116	27	46	29	33	55	28	0.5	PCR
Total							668	668								
***MSX1 rs12532***																PCR
6	Clarissa	2020	1	Brazil	Non-syndromic hypodontia	1	23	479	9	11	3	176	227	76	0.42	PCR
7	WANG H	2010	2	China	Non-syndromic hypodontia	1	198	207	31	101	66	40	110	57	0.46	PCR
8	Yu-Jin Seo	2013	2	Korea	Tooth agenesis	1	81	45	11	42	28	3	17	25	0.4	PCR
9	Gabriella	2013	3	Hungary	Non-syndromic hypodontia	1	192	260	98	85	9	120	111	29		PCR
10	Azza H. Al-Ani	2021	4	New Zealand	Non-syndromic hypodontia	1	61	297	27	24	10	134	142	21	0.38	PCR
11	Krisztina Mártha	2019	1	America	Non-syndromic hypodontia	1	97	99	50	37	10	45	42	12	0.37	PCR
12	Melissa Lancia	2020	1	Brazil	Non-syndromic hypodontia	1	110	157	43	52	15	75	61	21	0.39	PCR
Total							762	1544								
***PAX9 rs2073247***									**CaCC**	**CaCT**	**CaTT**	**ConCC**	**ConCT**	**ConTT**		
3	Isman	2013	1	Turkey	Non-syndromic hypodontia	2	200	114	19	98	83	14	53	47	0.4	PCR
1	Ahmed	2018	1	Jordan	Non-syndromic hypodontia	2	72	72	3	1	68	3	24	45	0.18	PCR
4	Peres	2005	1	Brazil	Non-syndromic hypodontia	2	102	106	43	46	13	60	43	3	0.35	PCR
4	Liu H	2012	2	China	Non-syndromic hypodontia	1	124	99	32	57	35	36	48	15	0.47	PCR
2	Gabriella	2013	3	Hungary	Non-syndromic hypodontia	1	192	260	51	111	30	98	121	41	0.44	PCR
8	Yu-Jin Seo	2013	2	Korea	Tooth agenesis	1	81	45	18	44	19	15	14	16	0.49	PCR
5	Yong chu Pan	2008	2	China	Non-syndromic hypodontia	1	102	116	27	40	35	26	57	33	0.48	PCR
Total							873	812								

Abbreviations and numbers: Ethnicity: 1 = Caucasian, 2 = Asian, 3 = Magyarok, 4 = Black race. Source of control: 1 = HB, hospital-based; 2 = PB, population-based; MAF, minor allele frequency.

### 3.2 Quantitative synthesis

In the analysis encompassing five studies on *PAX9 rs2073244* [21,23–26], seven studies on *MSX1 rs12532* [4,23,27–31], and seven analyses on *PAX9 rs2073247* [4,21–26], we found that partial models of *PAX9 rs2073247* and *PAX9 rs2073244* gene polymorphisms, along with race and origin, were associated with susceptibility to tooth loss.

**Figure 2:**
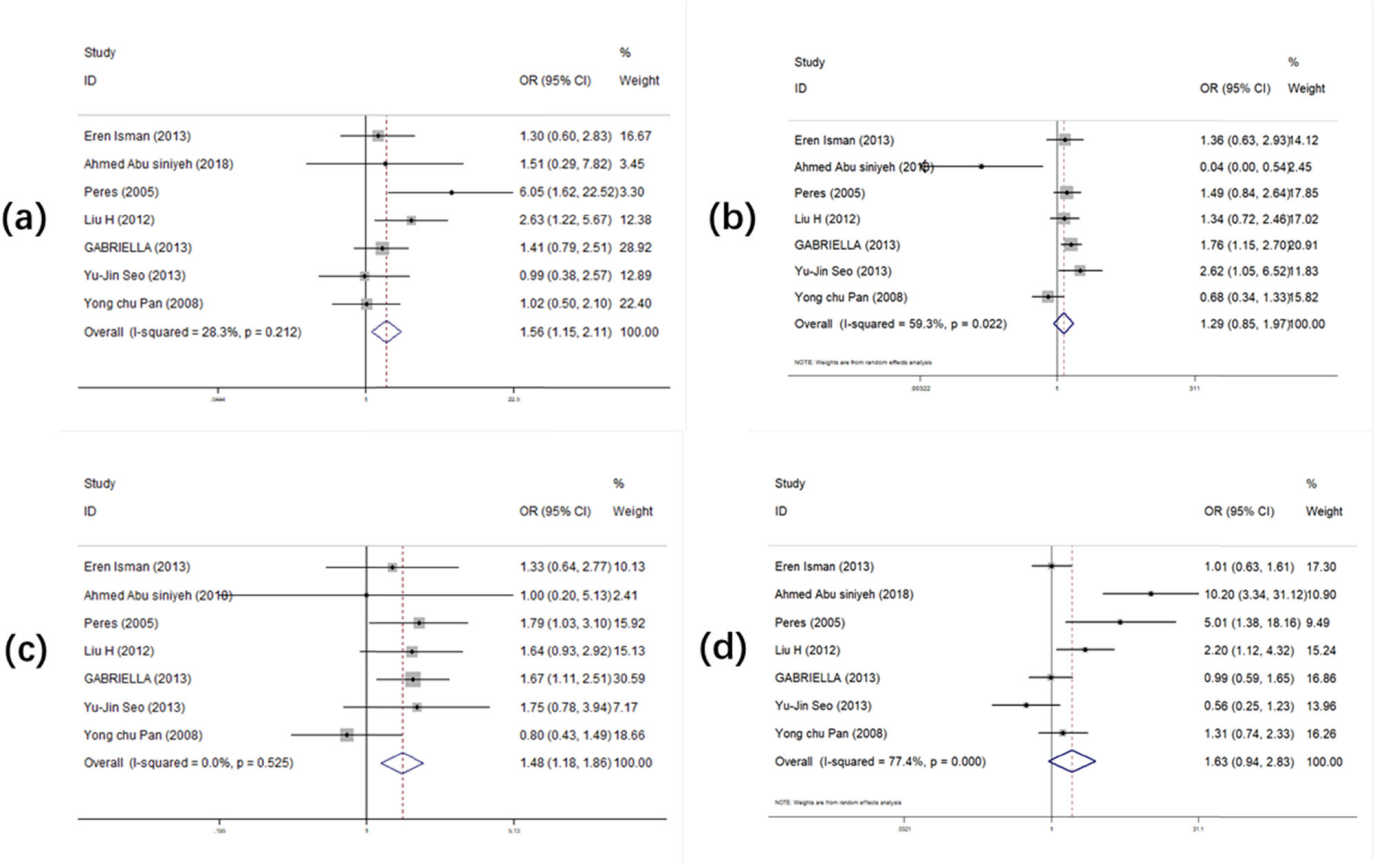
Forest plot of the statistical correlation between *PAX9 rs2073247* polymorphism and tooth agenesis susceptibility in all models: (a) TT vs CC, (b) CT vs CC, (c) CT + TT vs CC, and (d) TT vs CT + CC. Data were pooled ORs with 95% CIs determined using random-effects models or fixed-effects models according to *I* ^2^ values.

Table 2 and Figure 2 record the overall OR and 95% CI of the relationship between *PAX9 rs2073247* polymorphism and the risk of tooth loss. When analyzing the seven studies on *PAX9 rs2073247* in all four models, we found that two model of the polymorphism increased the susceptibility to tooth loss (TT vs CC: OR (95% CI) = 1.556 (1.147–2.112), *p* = 0.005; CT + TT vs CC: OR (95% CI) = 1.479 (1.176–1.862), *p* = 0.001). In the stratified analysis by ethnicity, we found that the Hungarian ethnic group had increased susceptibility to tooth loss with *PAX9 rs2073247* polymorphism (CT vs CC: OR (95% CI) = 1.763 (1.152–2.697), *p* = 0.009; CT + TT vs CC: OR (95% CI) = 1.672 (1.114–2.512), *p* = 0.013), while only one polymorphism model was associated with increased susceptibility to tooth loss in the Caucasian ethnic group (CT + TT vs CC: OR (95% CI) = 1.561 (1.023–2.381), *p* = 0.039). In the groups based on hospital origin and population origin, we found that the CT + TT vs CC model was associated with tooth loss in both (CT + TT vs CC: OR (95% CI) = 1.447 (1.100–1.903), *p* = 0.008; CT + TT vs CC: OR (95% CI) = 1.561 (1.023–2.381), *p* = 0.039).

**Table 2:** Stratified analyses of the *PAX9 rs2073247* polymorphism on tooth agenesis risk

Comparative model	No.	*Z*	*p*	OR (95% CI)	Heterogeneity			*Z*	Begg's test	*t*	Egger's test			FPRP statistical power					BEDP prior probability		
					Heterogeneity chi-squared	*p*	*I* ^2^					FPRP *p*-value	FPRP statistical power	0.250	0.100	0.010	0.001	0.0001	0.010	0.001	1 × 10^06^
**TT/CC**																					
**Overall**	7	2.840	0.005	1.556 (1.147–2.112)	8.370	0.212	28.30%	0.600	0.548	0.900	0.408	0.005	0.407	0.033	0.092	0.526	0.918	0.991	0.898	0.989	1.000
**Ethnicity**																					
Caucasian	3	1.520	0.128	2.143 (0.802–5.721)	4.000	0.135	50.10%	0.000	1.000	0.640	0.636	0.128	0.238	0.617	0.829	0.982	0.998	1.000	0.987	0.999	1.000
Asian	3	1.540	0.123	1.429 (0.908–2.249)	3.810	0.149	47.40%	0.000	1.000	–0.210	0.870	0.123	0.583	0.387	0.655	0.954	0.995	1.000	0.989	0.999	1.000
Magyarok	1	1.150	0.249	1.406 (0.787–2.511)	0.000	.	.%					0.249	0.587	0.561	0.793	0.977	0.998	1.000	0.992	0.999	1.000
**Source of control**																					
HB	4	1.930	0.054	1.420 (0.994–2.030)	3.810	0.283	21.20%	−0.340	1.000	−0.150	0.895	0.054	0.618	0.209	0.442	0.897	0.989	0.999	0.984	0.998	1.000
PB	3	1.520	0.128	2.143 (0.802–5.721)	4.000	0.135	50.10%	0.000	1.000	0.640	0.636								0.987	0.999	1.000
																					
**CT/CC**																					
**Overall**	7	1.200	0.231	1.293 (0.849–1.970 )	14.750	0.022	59.30%	0.900	0.368	−1.760	0.139	0.232	0.755	0.479	0.734	0.968	0.997	1.000	0.993	0.999	1.000
**Ethnicity**																					
Caucasian	3	0.190	0.852	0.903 (0.312–2.616 )	7.180	0.028	72.20%	1.040	0.296	−4.920	0.128	0.851	0.712	0.782	0.915	0.992	0.999	1.000	0.993	0.999	1.000
Asian	3	0.660	0.510	1.267 (0.626–2.565)	5.730	0.057	65.10%	0.000	1.000	0.780	0.579	0.511	0.681	0.692	0.871	0.987	0.999	1.000	0.994	0.999	1.000
Magyarok	1	2.610	0.009	1.763 (1.152–2.697)	0.000	.	.%					0.009	0.228	0.105	0.261	0.795	0.975	0.997	0.934	0.993	1.000
**Source of control**																					
HB	4	1.330	0.184	1.397 (0.853–2.290)	7.430	0.059	59.60%	−0.340	1.000	−0.190	0.869	0.185	0.611	0.476	0.731	0.968	0.997	1.000	0.991	0.999	1.000
PB	3	0.190	0.852	0.903 (0.312–2.616)	7.180	0.028	72.20%	1.040	0.296	−4.920	0.128	0.851	0.712	0.782	0.915	0.992	0.999	1.000	0.993	0.999	1.000
																					
**CT+TT/CC**																					
**Overall**	7	3.340	0.001	1.479 (1.176−1.862)	5.140	0.525	0.00%	1.200	0.230	−0.810	0.453	0.001	0.548	0.005	0.014	0.135	0.612	0.940	0.704	0.960	1.000
**Ethnicity**																					
Caucasian	3	2.060	0.039	1.561 (1.023−2.381)	0.700	0.705	0.00%	1.040	0.296	−1.700	0.338	0.039	0.427	0.214	0.449	0.900	0.989	0.999	0.977	0.998	1.000
Asian	3	1.300	0.195	1.279 (0.882–1.855)	3.480	0.176	42.50%	0.000	1.000	0.250	0.843	0.195	0.800	0.422	0.687	0.960	0.996	1.000	0.993	0.999	1.000
Magyarok	1	2.480	0.013	1.672 (1.114–2.512)	0.000	.	.%					0.013	0.301	0.117	0.285	0.814	0.978	0.998	0.951	0.995	1.000
**Source of control**																					
HB	4	2.650	0.008	1.447 (1.100–1.903)	4.360	0.225	31.20%	1.020	0.308	−0.420	0.717	0.008	0.602	0.039	0.109	0.574	0.932	0.993	0.938	0.994	1.000
PB	3	2.060	0.039	1.561 (1.023–2.381)	0.700	0.705	0.00%	1.040	0.296	−1.700	0.338	0.039	0.427	0.214	0.449	0.900	0.989	0.999			
																					
**TT/CT+CC**																					
**Overall**	7	1.740	0.081	1.630 (0.941–2.826)	26.520	0.000	77.40%	1.500	0.133	2.100	0.089	0.082	0.384	0.390	0.657	0.955	0.995	1.000	0.985	0.998	1.000
**Ethnicity**																					
Caucasian	3	1.520	0.128	3.487 (0.697–17.431)	17.690	0.000	88.70%	0.000	1.000	2.910	0.211	0.128	0.152	0.717	0.884	0.988	0.999	1.000	0.988	0.999	1.000
Asian	3	0.520	0.606	1.207 (0.590–2.470)	6.700	0.035	70.20%	0.000	1.000	–0.680	0.619	0.607	0.724	0.715	0.883	0.988	0.999	1.000	0.994	0.999	1.000
Magyarok	1	0.040	0.967	0.989 (0.592–1.652)	0.000	.	.%					0.966	0.934	0.756	0.903	0.990	0.999	1.000	0.996	1.000	1.000
**Source of control**																					
HB	4	0.560	0.577	1.150 (0.704–1.877)	7.300	0.063	58.90%	−0.340	1.000	−0.260	0.817	0.576	0.856	0.669	0.858	0.985	0.999	1.000	0.995	1.000	1.000
PB	3	1.520	0.128	3.487 (0.697–17.431)	17.690	0.000	88.70%	0.000	1.000	2.910	0.211	0.128	0.152	0.717	0.884	0.988	0.999	1.000	0.988	0.999	1.000

Abbreviations: OR, odds ratio; CI, confidence interval; PB, population-based; HB, hospital-based; FPRP, false positive report probability; BFDP, Bayesian false discovery probability. The results in bold represent there was statistically significant noteworthiness at 0.2 level by FPRP or 0.8 level by BFDP calculations.

**Figure 3:**
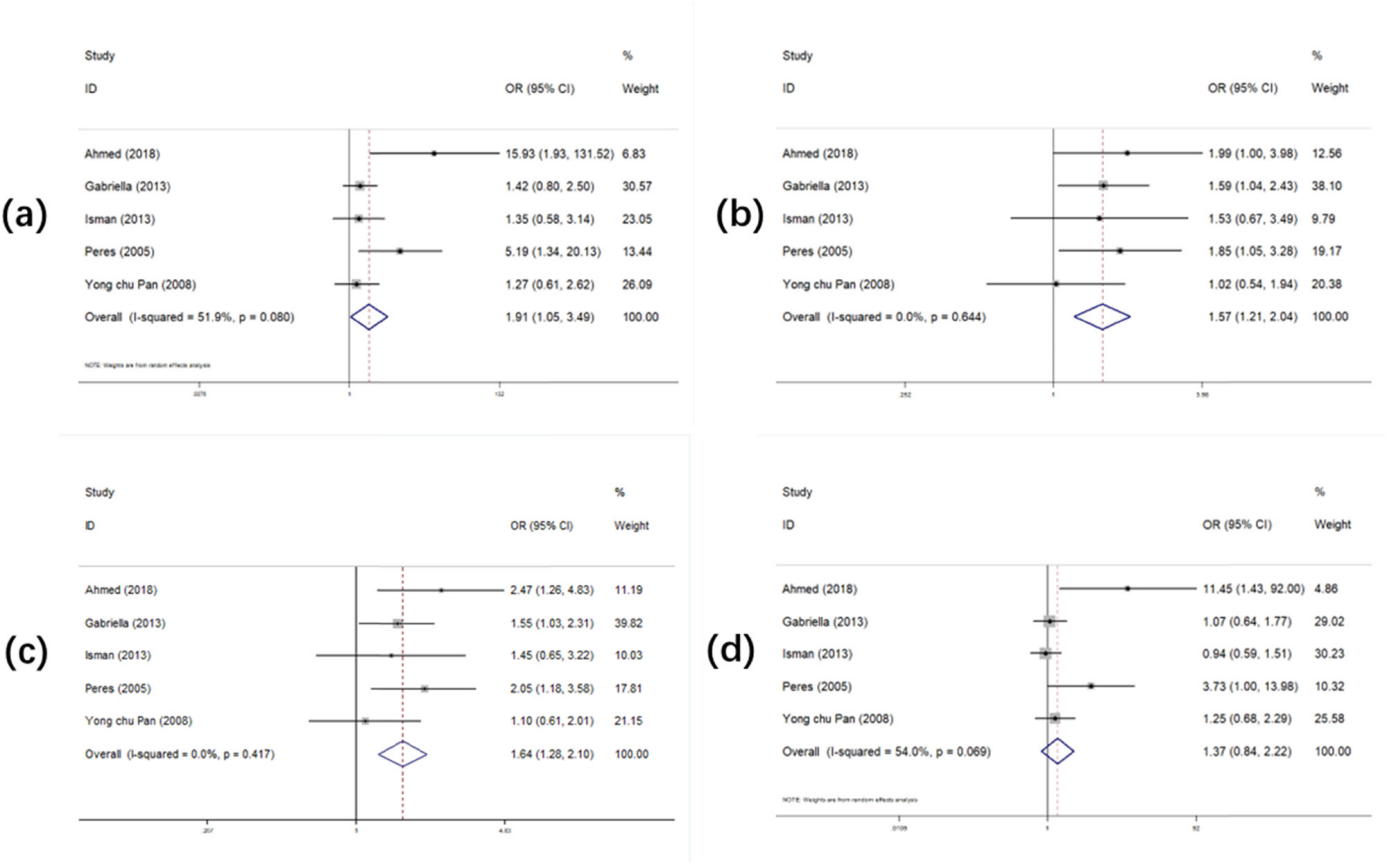
Forest plot of the statistical correlation between *PAX9 rs2073244* polymorphism and tooth agenesis susceptibility in all models: (a) GG vs AA, (b) AG vs AA, (c) AG + GG vs AA, and (d) GG vs AA + AG. Data were pooled ORs with 95% CIs determined using random-effects models or fixed-effects models according to *I* ^2^ value.

Table 3 and Figure 3 record the overall OR and 95% CI of the relationship between *PAX9 rs2073244* polymorphism and the risk of tooth loss in general. When analyzing the five studies on *PAX9 rs2073244* in all four models, we found that three of the models increased the susceptibility to tooth loss (GG vs AA: OR (95% CI) = 1.911 (1.046–3.493), *p* = 0.035; AG vs AA: OR (95% CI) = 1.569 (1.209–2.036), *p* = 0.001; AG + GG vs AA: OR (95% CI) = 1.637 (1.276–2.100), *p* = 0.000). In the AG vs AA and AG + GG vs AA models, we found that the risk of tooth loss was higher in the Caucasian race (AG vs AA: OR (95% CI) = 1.819 (1.234–2.679), *p* = 0.002; AG + GG vs AA: OR (95% CI) = 2.018 (1.387–2.938), *p* = 0.000) and the Hungarian race (AG vs AA: OR (95% CI) = 1.590 (1.042–2.425), *p* = 0.031; AG + GG vs AA: OR (95% CI) = 1.546 (1.034–2.312), *p* = 0.034). No model was found to be related to the risk of tooth loss in the hospital source. Two models were found to be related to the risk of tooth loss in the population source (AG vs AA: OR (95% CI) = 1.819 (1.234–2.679), *p* = 0.002; AG + GG vs AA: OR (95% CI) = 2.018 (1.387–2.938), *p* = 0.000).

**Table 3:** Stratified analyses of the *PAX9 rs2073244* polymorphism on tooth agenesis risk

Comparative model	No.	*Z*	*p*	OR (95% CI)	Heterogeneity			*Z*	Begg's test	*t*	Egger's test			FPRP statistical power					BEDP prior probability		
					Heterogeneity chi-squared	*p*	*I* ^2^					FPRP *p*-value	FPRP statistical power	0.250	0.100	0.010	0.001	1 × 10^−4^	0.010	0.001	0.000001
**GG/AA**																					
**Overall**	5	2.110	0.035	1.911 (1.046–3.493)	8.320	0.080	51.90%	1.710	0.086	4.080	0.027	0.035	0.216	0.330	0.596	0.942	0.994	0.999	0.974	0.997	1.000
**Ethnicity**																					
Caucasian	3	1.880	0.061	3.808 (0.942–15.404)	6.290	0.043	68.20%	1.040	0.296	6.020	0.105	0.061	0.096	0.656	0.851	0.984	0.998	1.000	0.985	0.998	1.000
Asia	1	0.640	0.525	1.266 (0.612–2.619)	0.000	.	.					0.525	0.676	0.700	0.875	0.987	0.999	1.000	0.994	0.999	1.000
Magyarok	1	1.200	0.231	1.416 (0.802–2.503)	0.000	.	.					0.231	0.579	0.545	0.783	0.975	0.998	1.000	0.992	0.999	1.000
**Source of control**																					
HB	2	1.330	0.182	1.357 (0.867–2.124)	0.060	0.811	0.00%	0.000	1.000	.	.	0.182	0.669	0.449	0.710	0.964	0.996	1.000	0.992	0.999	1.000
PB	3	1.880	0.061	3.808 (0.942–15.404)	6.290	0.043	68.20%	1.040	0.296	6.020	0.105	0.061	0.096	0.656	0.851	0.984	0.998	1.000	0.985	0.998	1.000
**AG/AA**																					
**Overall**	5	3.390	0.001	1.569 (1.209–2.036)	2.500	0.644	0.00%	–0.240	1.000	–0.140	0.894	0.001	0.368	0.006	0.017	0.159	0.656	0.950	0.652	0.950	0.999
**Ethnicity**																					
Caucasian	3	3.030	0.002	1.819 (1.234–2.679)	0.240	0.887	0.00%	0.000	1.000	–0.830	0.559	0.002	0.164	0.043	0.118	0.596	0.937	0.993	0.834	0.981	1.000
Asia	1	0.070	0.946	1.022 (0.538–1.942)	0.000	.	.%					0.947	0.879	0.764	0.906	0.991	0.999	1.000	0.995	0.999	1.000
Magyarok	1	2.150	0.031	1.590 (1.042–2.425)	0.000	.	.%					0.031	0.393	0.193	0.417	0.887	0.988	0.999	0.973	0.997	1.000
**Source of control**																					
HB	2	1.840	0.065	1.392 (0.979–1.979)	1.270	0.260	21.20%	0.000	1.000	.	.	0.065	0.661	0.229	0.471	0.907	0.990	0.999	0.986	0.999	1.000
PB	3	3.030	0.002	1.819 (1.234–2.679)	0.240	0.887	0.00%	0.000	1.000	–0.830	0.559	0.002	0.164	0.043	0.118	0.596	0.937	0.993	0.834	0.981	1.000
**AG+GG/AA**																					
**Overall**	5	3.880	0.000	1.637 (1.276–2.100)	3.920	0.417	0.00%	0.240	0.806	0.230	0.832	0.000	0.246	0.001	0.004	0.041	0.299	0.810	0.259	0.779	0.997
**Ethnicity**																					
Caucasian	3	3.670	0.000	2.018 (1.387–2.938)	1.010	0.603	0.00%	0.000	1.000	–0.680	0.620	0.000	0.061	0.012	0.035	0.288	0.803	0.976	0.429	0.884	0.999
Asia	1	0.330	0.744	1.104 (0.608–2.006)	0.000	.	.%					0.745	0.843	0.726	0.888	0.989	0.999	1.000	0.995	1.000	1.000
Magyarok	1	2.120	0.034	1.546 (1.034–2.312)	0.000	.	.%					0.034	0.442	0.187	0.408	0.884	0.987	0.999	0.975	0.998	1.000
**Source of control**																					
HB	2	1.950	0.051	1.393 (0.998–1.943)	0.840	0.360	0.00%	0.000	1.000	.	.	0.051	0.669	0.186	0.407	0.883	0.987	0.999	0.984	0.998	1.000
PB	3	3.670	0.000	2.018 (1.387–2.938)	1.010	0.603	0.00%	0.000	1.000	–0.680	0.620	0.000	0.061	0.012	0.035	0.288	0.803	0.976	0.429	0.884	0.999
**GG/AG+AA**																					
**Overall**	5	1.250	0.211	1.365 (0.838–2.225)	8.700	0.069	54.00%	2.200	0.027	20.130	0.000	0.212	0.647	0.496	0.747	0.970	0.997	1.000	0.992	0.999	1.000
**Ethnicity**																					
Caucasian	3	1.320	0.186	2.674 (0.622–11.496)	8.620	0.013	76.80%	1.040	0.296	40.830	0.016	0.186	0.219	0.719	0.885	0.988	0.999	1.000	0.989	0.999	1.000
Asia	1	0.720	0.472	1.249 (0.682–2.286)	0.000	.	.%					0.471	0.724	0.661	0.854	0.985	0.998	1.000	0.994	0.999	1.000
Magyarok	1	0.260	0.798	1.068 (0.645–1.771)	0.000	.	.%					0.799	0.906	0.726	0.888	0.989	0.999	1.000	0.996	1.000	1.000
**Source of control**																					
HB	2	0.660	0.510	1.139 (0.773–1.678)	0.150	0.698	0.00%	0.000	1.000	.	.	0.510	0.918	0.625	0.833	0.982	0.998	1.000	0.996	1.000	1.000
PB	3	1.320	0.186	2.674 (0.622–11.496)	8.620	0.013	76.80%	1.040	0.296	40.830	0.016	0.186	0.219	0.719	0.885	0.988	0.999	1.000	0.989	0.999	1.000

Abbreviations: OR, odds ratio; CI, confidence interval; PB, population-based; HB, hospital-based; FPRP, false positive report probability; BFDP, Bayesian false discovery probability. The results in bold represent there was statistically significant noteworthiness at 0.2 level by FPRP or 0.8 level by BFDP calculations.

**Figure 4:**
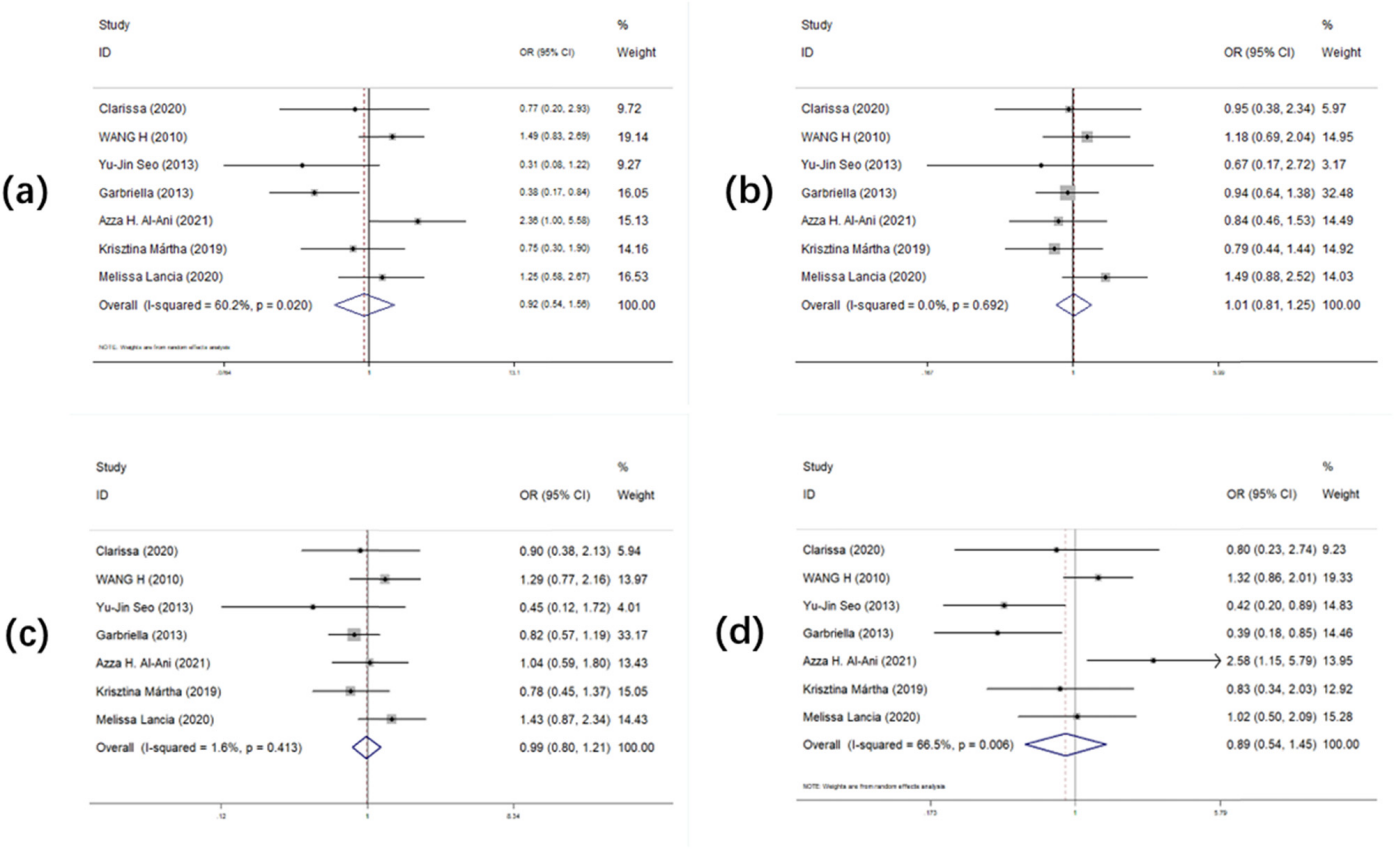
Forest plot of the statistical correlation between *MSX1 rs12532* polymorphism and tooth agenesis susceptibility in all models: (a) GG vs AA, (b) AG vs AA, (c) AG + GG vs AA, and (d) GG vs AA + AG. Data were pooled ORs with 95% CIs determined using random-effects models or fixed-effects models according to *I* ^2^ values.

Table 4 and Figure 4 record the overall OR and 95% CIs for the association of *MSX1 rs12532* polymorphism with tooth loss risk. In the analysis of seven studies on *MSX1 rs12532* in all four models, we did not find any model associated with increased sensitivity to tooth loss, but in the analysis based on race, we found that Hungarian race was associated with increased tooth loss risk in two models (GG vs AA: OR (95% CI) = 0.380 (0.172–0.841), *p* = 0.017; GG vs AG + AA: OR (95% CI) = 0.392 (0.181–0.848), *p* = 0.017). Only one model was found to be associated with tooth loss in Black race (GG vs AG + AA: OR (95% CI) = 2.577 (1.146–5.794), *p* = 0.022).

**Table 4:** Stratified analyses of the *MSX1 rs12532* polymorphism on tooth agenesis risk

Comparative model	No.	*Z*	*p*	OR (95% CI)	Heterogeneity			*Z*	Begg's test	*t*	Egger's test			FPRP statistical power					BEDP prior probability		
					Heterogeneity chi-squared	*p*	*I* ^2^					FPRP *p*-value	FPRP statistical power	0.250	0.100	0.010	0.001	0.0001	0.010	0.001	0.000001
**GG/AA**																					
**Overall**		0.320	0.746	0.916 (0.538–1.560)	15.080	0.020	60.20%	0.900	0.368	−1.230	0.275	0.747	0.879	0.718	0.884	0.988	0.999	1.000	0.995	1.000	1.000
**Ethnicity**																					
Caucasian		0.120	0.906	0.968 (0.566–1.656)	0.820	0.663	0.00%	0.000	1.000	–1.040	0.486	0.905	0.913	0.748	0.899	0.990	0.999	1.000	0.996	1.000	1.000
Asian		0.340	0.737	0.768 (0.165–3.580)	4.290	0.038	76.70%	0.000	1.000	.	.	0.737	0.571	0.795	0.921	0.992	0.999	1.000	0.992	0.999	1.000
Magyarok		2.390	0.017	0.380 (0.172–0.841)	0.000	.	.%					0.017	0.083	0.381	0.649	0.953	0.995	1.000	0.963	0.996	1.000
Black race		1.960	0.050	2.363 (1.001–5.580)	0.000	.	.%					0.050	0.150	0.499	0.749	0.970	0.997	1.000	0.980	0.998	1.000
**Source of control**																					
HB		0.320	0.746	0.916 (0.538–1.560)	15.080	0.020	60.20%	0.900	0.368	–1.230	0.275	0.747	0.879	0.718	0.884	0.988	0.999	1.000	0.995	1.000	1.000
PB																					
																					
**AG/AA**																					
**Overall**		0.070	0.943	1.008 (0.811–1.253)	3.890	0.692	0.00%	0.600	0.548	–0.600	0.575	0.943	1.000	0.739	0.895	0.989	0.999	1.000	0.998	1.000	1.000
**Ethnicity**																					
Caucasian		0.510	0.610	1.098 (0.766–1.575)	2.510	0.284	20.50%	0.000	1.000	–0.510	0.699	0.612	0.955	0.658	0.852	0.984	0.998	1.000	0.996	1.000	1.000
Asian		0.360	0.721	1.095 (0.664–1.807)	0.550	0.460	0.00%	0.000	1.000	.	.	0.723	0.891	0.709	0.879	0.988	0.999	1.000	0.996	1.000	1.000
Magyarok		0.320	0.745	0.938 (0.636–1.383)	0.000	.	.%					0.747	0.958	0.701	0.875	0.987	0.999	1.000	0.996	1.000	1.000
Black race		0.580	0.565	0.839 (0.461–1.526)	0.000	.	.%					0.565	0.774	0.686	0.868	0.986	0.999	1.000	0.995	0.999	1.000
**Source of control**																					
HB		0.070	0.943	1.008 (0.811–1.253 )	3.890	0.692	0.00%	0.600	0.548	–0.600	0.575	0.943	1.000	0.739	0.895	0.989	0.999	1.000	0.998	1.000	1.000
PB																					
**GG+AG/AA**																					
**Overall**		0.120	0.904	0.987 (0.804–1.213)	6.100	0.413	1.60%	0.900	0.368	–0.600	0.576	0.901	1.000	0.730	0.890	0.989	0.999	1.000	0.998	1.000	1.000
**Ethnicity**																					
Caucasian		0.360	0.716	1.065 (0.759–1.495)	2.620	0.269	23.80%	0.000	1.000	–0.540	0.683	0.716	0.976	0.688	0.868	0.986	0.999	1.000	0.997	1.000	1.000
Asian		0.160	0.871	0.924 (0.355–2.402)	2.050	0.152	51.30%	0.000	1.000	.	.	0.871	0.748	0.777	0.913	0.991	0.999	1.000	0.993	0.999	1.000
Magyarok		1.030	0.304	0.822 (0.566–1.194)	0.000	.	.%					0.303	0.864	0.513	0.760	0.972	0.997	1.000	0.995	0.999	1.000
Black race		0.120	0.903	1.035 (0.595–1.802)	0.000	.	.%					0.903	0.905	0.750	0.900	0.990	0.999	1.000	0.995	1.000	1.000
**Source of control**																					
HB		0.120	0.904	0.987 (0.804–1.213)	6.100	0.413	1.60%	0.900	0.368	–0.600	0.576	0.901	1.000	0.730	0.890	0.989	0.999	1.000	0.998	1.000	1.000
PB																					
																					
**GG/AG+AA**																					
**Overall**		0.480	0.632	0.888 (0.544–1.447)	17.930	0.006	66.50%	0.300	0.764	–0.790	0.465	0.633	0.875	0.685	0.867	0.986	0.999	1.000	0.996	1.000	1.000
**Ethnicity**																					
Caucasian		0.340	0.732	0.915 (0.552–1.518)	0.180	0.912	0.00%	0.000	1.000	–1.730	0.333	0.731	0.890	0.711	0.881	0.988	0.999	1.000	0.996	1.000	1.000
Asian		0.440	0.658	0.778 (0.257–2.362)	6.730	0.009	85.20%	0.000	1.000	.	.	0.658	0.607	0.765	0.907	0.991	0.999	1.000	0.993	0.999	1.000
Magyarok		2.380	0.017	0.392 (0.181–0.848)	0.000	.	.%					0.017	0.089	0.370	0.638	0.951	0.995	0.999	0.963	0.996	1.000
Black race		2.290	0.022	2.577 (1.146–5.794)	0.000	.	.%					0.022	0.095	0.410	0.675	0.958	0.996	1.000	0.968	0.997	1.000
**Source of control**																					
HB		0.480	0.632	0.888 (0.544–1.447)	17.930	0.006	66.50%	0.300	0.764	–0.790	0.465	0.633	0.875	0.685	0.867	0.986	0.999	1.000	0.996	1.000	1.000
PB																					

Abbreviations: OR, odds ratio; CI, confidence interval; PB, population-based; HB, hospital-based; FPRP, false positive report probability; BFDP, Bayesian false discovery probability. The results in bold represent there was statistically significant noteworthiness at 0.2 level by FPRP or 0.8 level by BFDP calculations.

### 3.3 Publication bias

**Figure 5:**
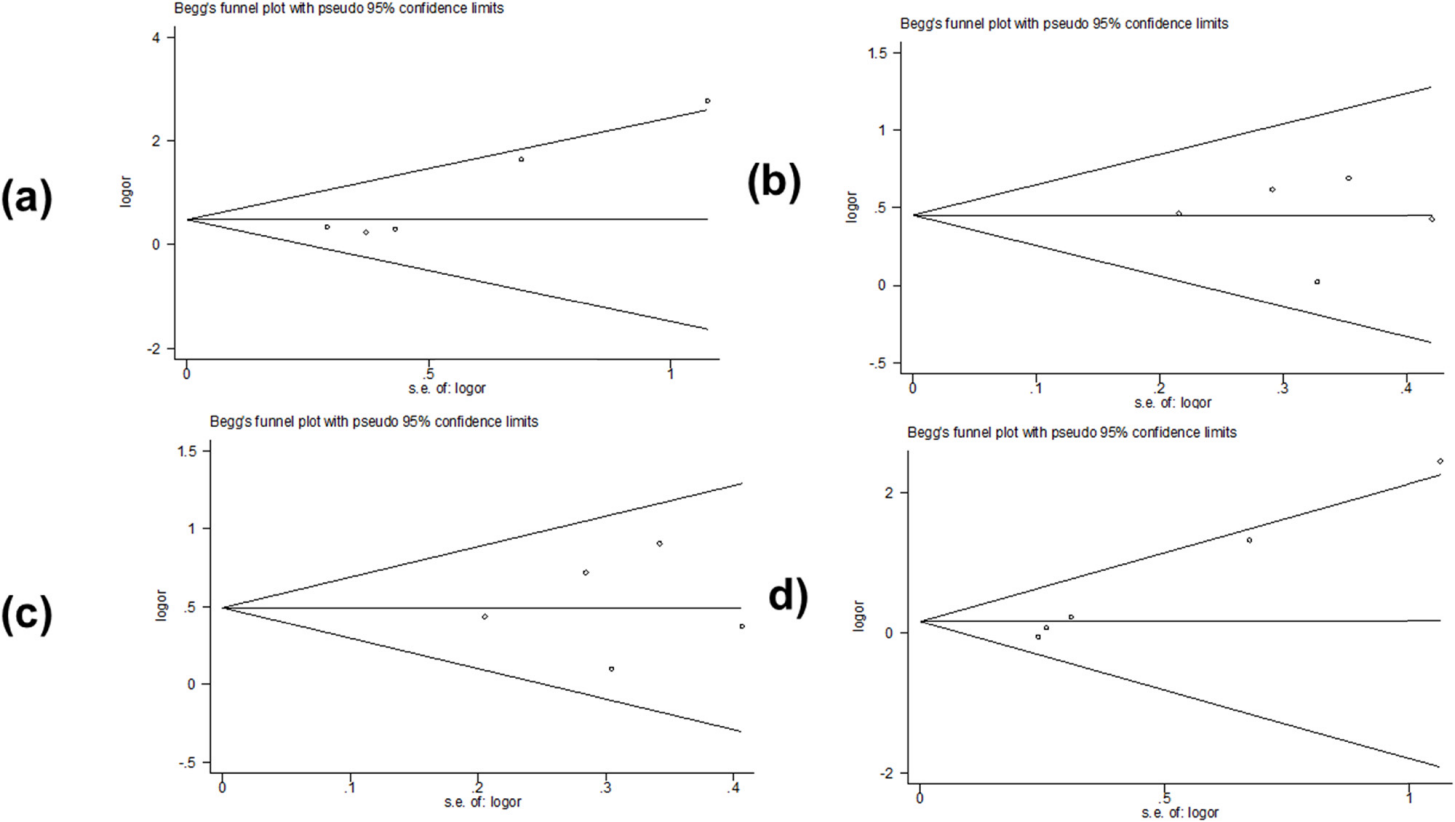
Begg’s funnel plot assessed the risk of *PAX9 rs2073244* publication bias in the meta-analysis: (a) GG vs AA, (b) AG vs AA, (c) GG + AG vs AA, and (d) GG vs AG + AA.

**Figure 6:**
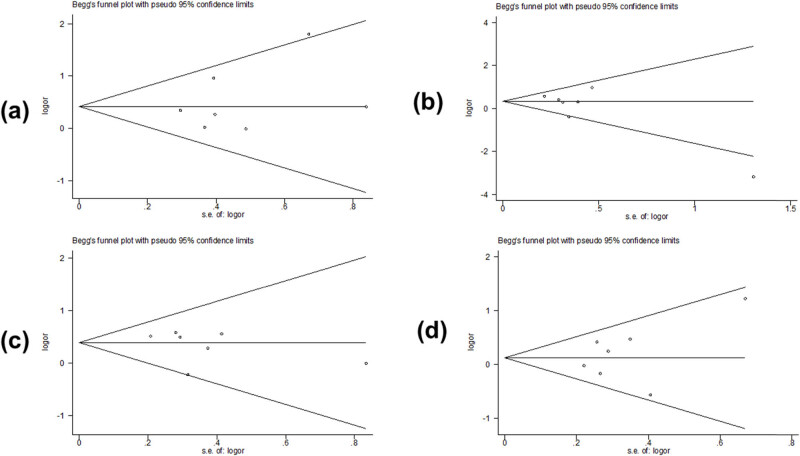
Begg’s funnel plot assessed the risk of *PAX9 rs2073247* publication bias in the meta-analysis: (a) TT vs CC, (b) CT vs CC, (c) CT + TT vs CC, and (d) TT vs CT + CC.

**Figure 7:**
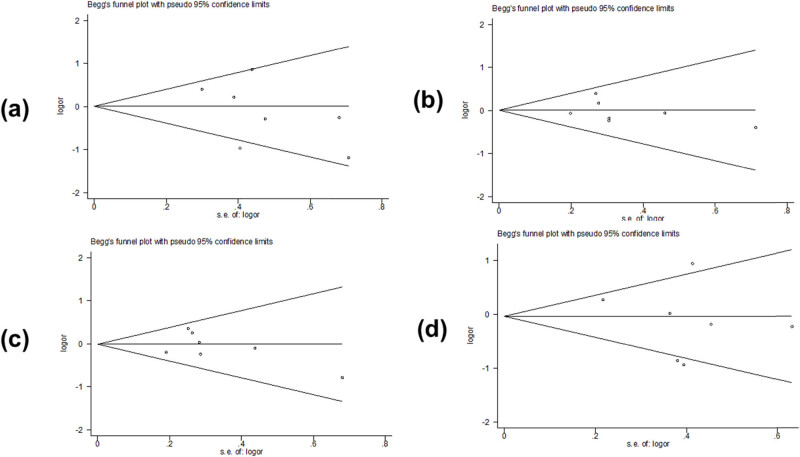
Begg’s funnel plot assessed the risk of *MSX1 rs12532* publication bias in the meta-analysis: (a) GG vs AA, (b) AG vs AA, (c) GG + AG vs AA, and (d) GG vs AG + AA.

Begg’s test and Egger’s test were used to assess potential publication bias. Partial results indicated publication bias in the two models of *PAX9 rs2073244* (GG vs AA: *p* = 0.027) and (GG vs AG + AA: *p* = 0.000) (Table 3 and Figure 5). However, polymorphic results of *PAX9 rs2073247* and *MSX1 rs12532* showed no evidence of publication bias (Table 2, Figure 6 and Table 4, Figure 7).

### 3.4 Sensitivity analysis

**Figure 8:**
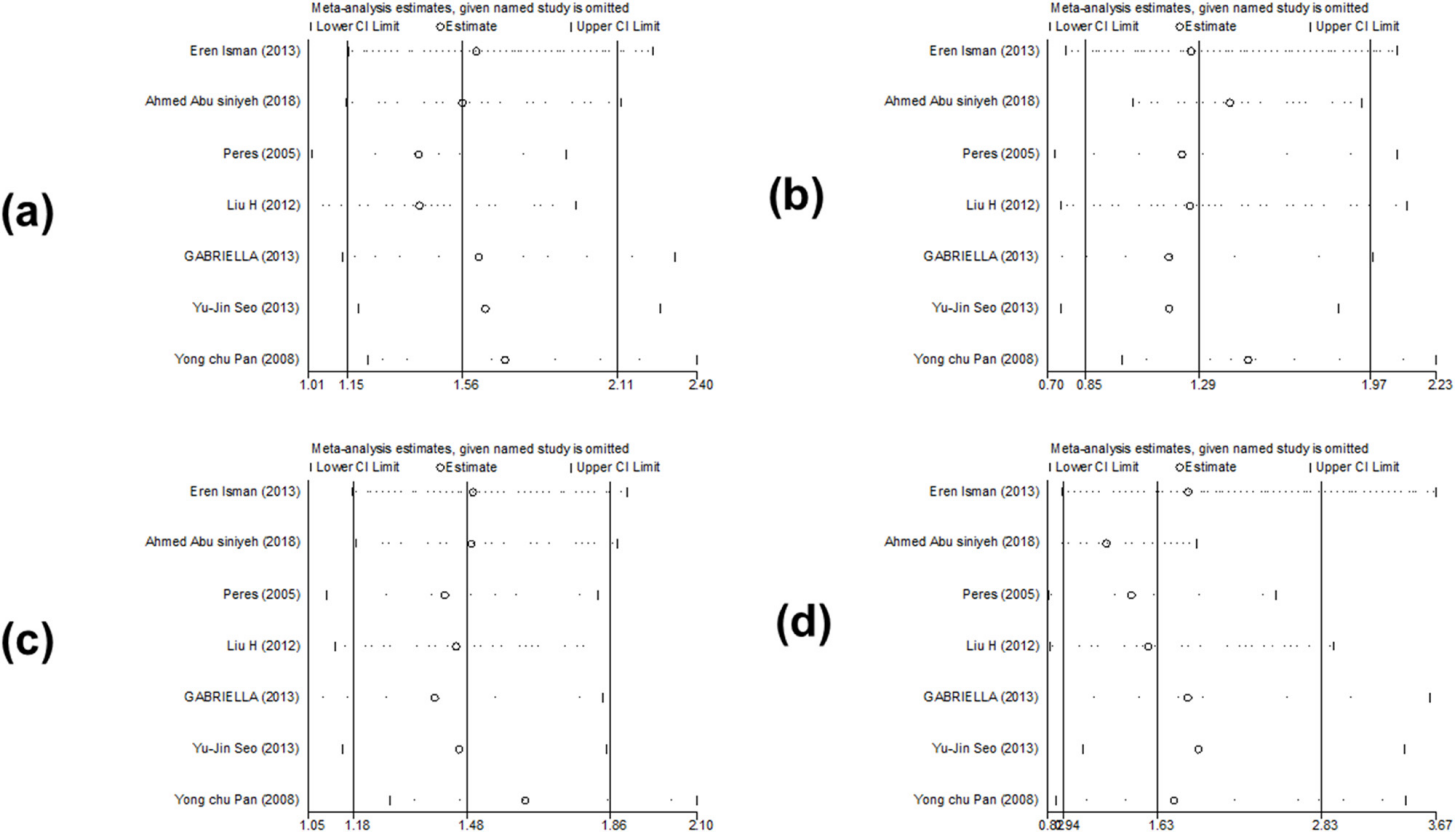
Sensitivity analysis of the association between polymorphism of *PAX9 rs2073247* and risk of tooth agenesis: (a) TT vs CC, (b) CT vs CC, (c) CT + TT vs CC, and (d) TT vs CT + CC.

**Figure 9:**
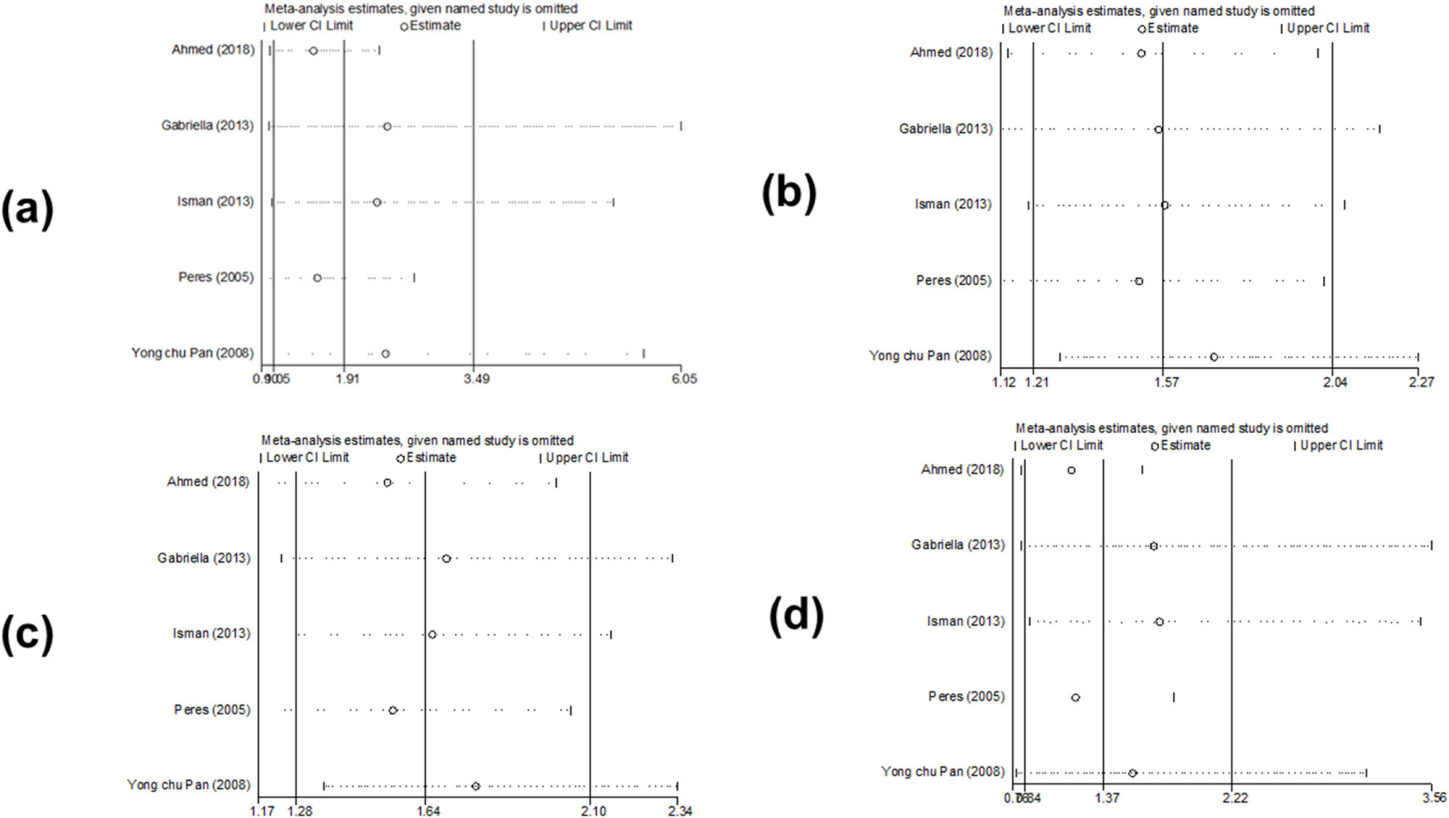
Sensitivity analysis of the association between polymorphism of *PAX9 rs2073244* and risk of tooth agenesis: (a) GG vs AA, (b) AG vs AA, (c) GG + AG vs AA, and (d) GG vs AG + AA.

**Figure 10:**
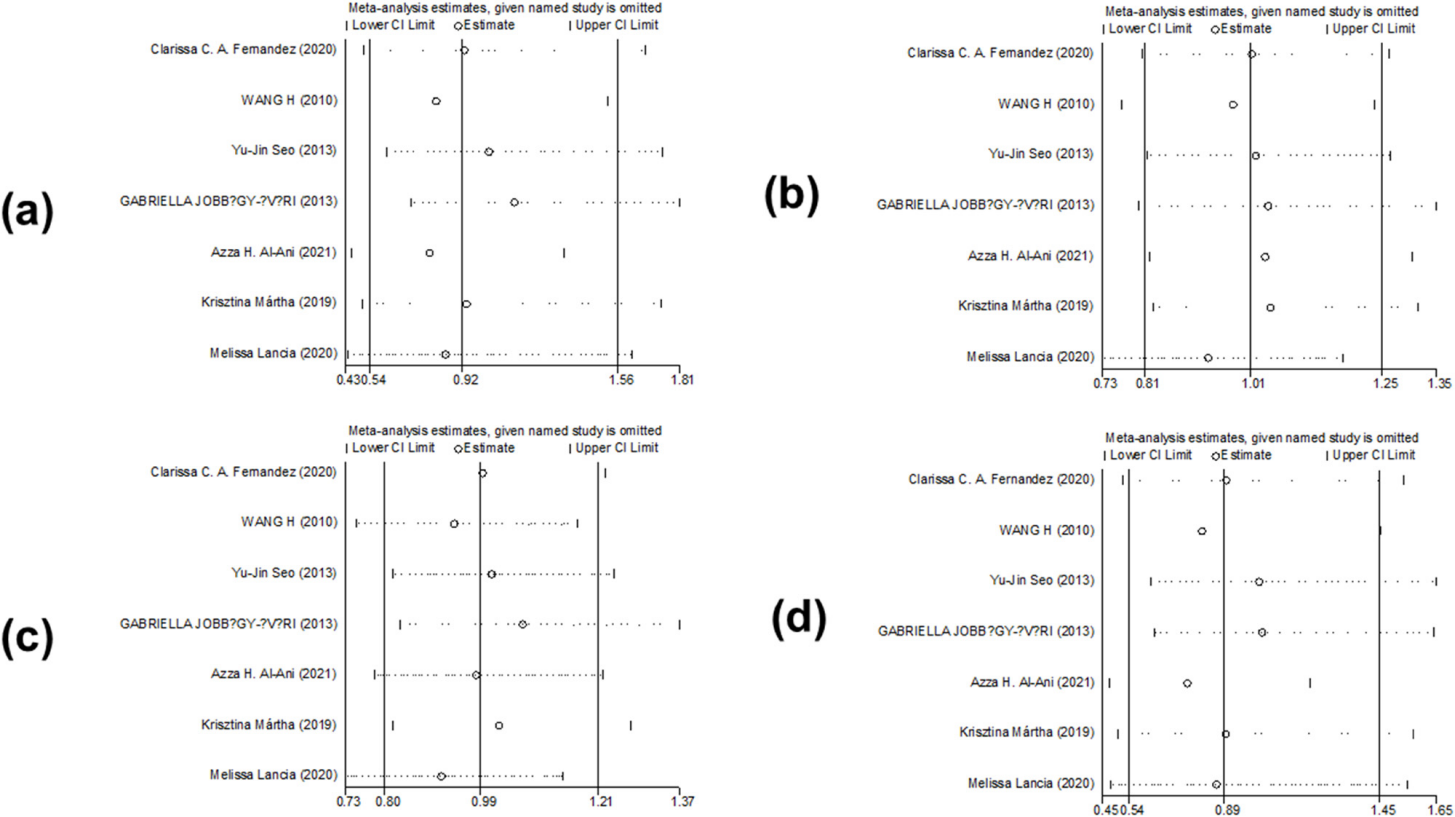
Sensitivity analysis of the association between polymorphism of *MSX1 rs12532* and risk of tooth agenesis: (a) GG vs AA, (b) AG vs AA, (c) GG + AG vs AA, and (d) GG vs AG + AA.

We did not find any change in the results of *PAX9 rs2073247*, *PAX9 rs2073244*, and *MSX1 rs12532* in the sensitivity analysis comparison. This suggests that the results of *PAX9 rs2073247*, *PAX9 rs2073244*, and *MSX1 rs12532* were statistically stable and reliable. Meta-regression analysis found that the relationship among publication time, race, and source of control did not affect the stability of the comprehensive results (Figures 8–10).

### 3.5 FPRP and BFDP test

The FPRP values of statistical power for significant findings about the *PAX9 rs2073247*, *PAX9 rs2073244*, and *MSX1 rs12532* polymorphism are shown in Tables 2–4. In Table 2, based on FPRP analyses, almost all models of *PAX9 rs2073247* polymorphism were found to be not significant by FPRP estimation at the OR of 1.5 with the prior probability of 0.25 and 0.1, and were not significant by BFDP test at the OR of 1.5 with the prior probability of 0.01, 0.001, and 0.000001. In Table 3, FPRP analyses revealed that half of the models of *PAX9 rs2073244* polymorphism were found to be noteworthy at an OR of 1.5 with the prior probability of 0.25 and 0.1. However, most of *PAX9 rs2073244* polymorphism models did not meet the criteria for significance according to BFDP test at OR of 1.5 with the prior probability of 0.01, 0.001, and 0.000001. In Table 4, half of the models of *MSX1 rs12532* polymorphism were found to be noteworthy by FPRP estimation at an OR of 1.5 with the prior probability of 0.25 and 0.1, but were not noteworthy by BFDP test at an OR of 1.5 with the prior probability of 0.01, 0.001, and 0.000001.

## 4 Discussion

Tooth loss is a significant oral health issue that can affect individuals of all ages. It can result from various factors such as dental decay, periodontal disease, trauma, or developmental disorders. These factors include trauma-induced injury to the tooth bud, viral infections, and maternal exposure to rubella or chemical drugs during pregnancy [32]. However, with the deepening understanding of molecular genetics, numerous studies have indicated a significant genetic component to tooth loss. The *PAX* gene family is named for its conserved paired domains. Based on the composition of the domains and the homology of the sequences, the gene family comprises four subfamilies: *PAX1/9 (PAX1, PAX9)*, *PAX2/5/8 (PAX2, PAX5, PAX8)*, *PAX3/7 (PAX3, PAX7)*, and *PAX4/6 (PAX4, PAX6)*. Among these, PAX9 is crucial in regulating the development of the maxillofacial region, tail, limb buds, esophagus, teeth, and pharynx, playing an upstream regulatory role in tooth development. Studies have suggested that mutations in *PAX9* and *MSX1* during the bud stage can prevent the mesenchymal cells from properly aggregating around the epithelial tooth bud, leading to impaired tooth development [33,34]. Meanwhile, *in vitro* biochemical assays have shown that *MSX1* and *PAX9* cooperate to activate the *BMP4* gene promoter, and the *BMP4* signal in mesenchymal cells is involved in the induction of the enamel knot, a transient signaling center in epithelial tissue that guides the next stage of tooth development. Therefore, it is hypothesized that polymorphisms in *PAX9* and *MSX1* may increase the risk of tooth agenesis in individuals. A review of the literature revealed that two similar studies have been previously published [35,36]. Compared to these studies, our research includes the following additions: (1) several articles published post-2014 have been incorporated and (2) compared and analyzed the subgroups of ethnic origin and control source. For this study, the results of judging the correlation between *PAX9*, *MSX1* and tooth loss risk through comprehensive analysis of all eligible case–control studies are controversial.

In the five studies on *PAX9 rs2073244*, seven studies on *MSX1 rs12532*, and seven studies on *PAX9 rs2073247*, we found that *PAX9 rs2073244* and *PAX9 rs2073247* were statistically associated with the risk of tooth loss. The G allele of *PAX9 rs2073244* and G carriers (AG + GG) may increase the risk of tooth loss and may be a risk factor for tooth loss. Similarly, the T allele of *PAX9 rs2073247* and T carriers (CT + TT) were positively associated with the risk of tooth loss, but we did not observe an association between *MSX1 rs12532* and tooth loss.

In our current meta-analysis, focusing on ethnic level analysis of *PAX9 rs2073244* and *PAX9 rs2073247*, we observed an increases risk of tooth loss among G carriers of *PAX9 rs2073244* in Caucasian and Hungarian populations, as well as a heightened risk of tooth loss among T carriers of *PAX9 rs2073247* in the same population. The risk of tooth loss increases in both populations in the same model, which may be due to the following reasons: (1) *PAX9 rs2073247* and *PAX9 rs2073244* are both polymorphic mutation sites of *PAX9*, which mainly functions in tooth development, and the mutated sites are mainly related to the position and number of tooth loss [37]; (2) the Caucasian population, being widely distributed, has historically undergone genetic intermixture with the early Hungarian population [38], so *PAX9* mutations may have similar regulatory effects on the development of tooth germ in these two populations; (3) some objective factors, such as sample size, selection bias, and inclusion or exclusion criteria, may also have some influence. Therefore, confirming this phenomenon requires a large-scale population study and multiple analyses to further prove the relationship between *PAX9 rs2073244*, *PAX9 rs2073247* gene polymorphisms and the risk of tooth loss.

In this meta-analysis, several limitations should be acknowledged. First, despite comprehensive database searches, the body of research concerning oral tooth loss remains limited, with sample sizes still relatively small, particularly regarding *PAX9 rs2073244* and *MSX1 rs12532*. Second, mutations at different sites of *PAX9* can cause dental agenesis with different manifestations, including the location and number of missing teeth, as well as other conditions such as maxillofacial deformities and cancer. Variations in diagnosis by different clinicians may lead to heterogeneity. For example, whether to include the loss of the third molars into the scope of tooth loss, corresponding subgroup analysis can be conducted for further study in order to eliminate the heterogeneity caused by clinical diagnostic criteria. Moreover, the control source for MSX1 rs12532 is relatively singular precluding that cross-sectional comparisons between control sources cannot be achieved. Finally, *MSX1* can interact with *PAX9* to regulate tooth development [39], and research on *MSX1* and *PAX9* may provide a deeper understanding of the impact of genetic polymorphisms on the risk of tooth loss.

## 5 Conclusion

Understanding the mechanisms of gene mutations will enable clinicians and human geneticists to open up new lines of thought for alternative treatment research in the future. Gene polymorphisms of *PAX9 rs2073247* (TT vs CC, CT + TT vs CC) and *PAX9 rs2073244* (GG vs AA, AG vs AA, AG + GG vs AA) may increase the risk of non-syndromic hypodontia, and the susceptibility is more obvious in Caucasians and Hungarians. However, no statistically significant association was found between *MSX1 rs12532* and the risk of tooth loss. *PAX9* polymorphisms might be involved in the pathogenesis of tooth loss. Due to the limited number of studies published in this field, the available evidence is still limited, so we emphasize the need for large studies with sufficient case numbers and appropriate control of confounding factors to yield robust findings.

## References

[1] Boeira Junior BR, Echeverrigaray S. Dentistry and molecular biology: a promising field for tooth agenesis management. Tohoku J Exp Med. 2012;226(4):243–9. 10.1620/tjem.226.243 22452934

[2] Yu M, Wong SW, Han D, Cai T. Genetic analysis: WNT and other pathways in nonsyndromic tooth agenesis. Oral Dis. 2019;25(3):646–51. 10.1111/odi.12931 PMC6318069 29969831

[3] Shastry BS. SNPs: impact on gene function and phenotype. Methods Mol Biol. 2009;578:3–22. 10.1007/978-1-60327-411-1_1 19768584

[4] Seo YJ, Park JW, Kim YH, Baek SH. Associations between the risk of tooth agenesis and single-nucleotide polymorphisms of MSX1 and PAX9 genes in nonsyndromic cleft patients. Angle Orthod. 2013;83(6):1036–42. 10.2319/020513-104.1 PMC8722836 23718693

[5] Vastardis H, Karimbux N, Guthua SW, Seidman JG, Seidman CE. A human MSX1 homeodomain missense mutation causes selective tooth agenesis. Nat Genet. 1996;13(4):417–21. 10.1038/ng0896-417 8696335

[6] Nieminen P, Arte S, Tanner D, Paulin L, Alaluusua S, Thesleff I, et al. Identification of a nonsense mutation in the PAX9 gene in molar oligodontia. Eur J Hum Genet: EJHG. 2001;9(10):743–6. 10.1038/sj.ejhg.5200715 11781684

[7] Pinho T, Silva-Fernandes A, Bousbaa H, Maciel P. Mutational analysis of MSX1 and PAX9 genes in Portuguese families with maxillary lateral incisor agenesis. Eur J Orthod. 2010;32(5):582–8. 10.1093/ejo/cjp155 20660504

[8] Mostowska A, Biedziak B, Trzeciak WH. A novel mutation in PAX9 causes familial form of molar oligodontia. Eur J Hum Genet: EJHG. 2006;14(2):173–9. 10.1038/sj.ejhg.5201536 16333316

[9] Paixão-Côrtes VR, Braga T, Salzano FM, Mundstock K, Mundstock CA, Bortolini MC. PAX9 and MSX1 transcription factor genes in non-syndromic dental agenesis. Arch Oral Biol. 2011;56(4):337–44. 10.1016/j.archoralbio.2010.10.020 21111400

[10] Boeira Junior BR, Echeverrigaray S. Polymorphism in the MSX1 gene in a family with upper lateral incisor agenesis. Arch Oral Biol. 2012;57(10):1423–8. 10.1016/j.archoralbio.2012.04.008 22591773

[11] Nakatomi M, Wang XP, Key D, Lund JJ, Turbe-Doan A, Kist R, et al. Genetic interactions between Pax9 and Msx1 regulate lip development and several stages of tooth morphogenesis. Dev Biol. 2010;340(2):438–49. 10.1016/j.ydbio.2010.01.031 20123092

[12] Satokata I, Maas R. Msx1 deficient mice exhibit cleft palate and abnormalities of craniofacial and tooth development. Nat Genet. 1994;6(4):348–56. 10.1038/ng0494-348 7914451

[13] Jumlongras D, Lin JY, Chapra A, Seidman CE, Seidman JG, Maas RL, et al. A novel missense mutation in the paired domain of PAX9 causes non-syndromic oligodontia. Hum Genet. 2004;114(3):242–9. 10.1007/s00439-003-1066-6 14689302

[14] Peters H, Neubüser A, Kratochwil K, Balling R. Pax9-deficient mice lack pharyngeal pouch derivatives and teeth and exhibit craniofacial and limb abnormalities. Genes Dev. 1998;12(17):2735–47. 10.1101/gad.12.17.2735 PMC317134 9732271

[15] Ogawa T, Kapadia H, Feng JQ, Raghow R, Peters H, D’Souza RN. Functional consequences of interactions between Pax9 and Msx1 genes in normal and abnormal tooth development. J Biol Chem. 2006;281(27):18363–9. 10.1074/jbc.M601543200 16651263

[16] Laurikkala J, Kassai Y, Pakkasjärvi L, Thesleff I, Itoh N. Identification of a secreted BMP antagonist, ectodin, integrating BMP, FGF, and SHH signals from the tooth enamel knot. Dev Biol. 2003;264(1):91–105. 10.1016/j.ydbio.2003.08.011 14623234

[17] Bonczek O, Balcar VJ, Šerý O. PAX9 gene mutations and tooth agenesis: a review. Clin Genet. 2017;92(5):467–76. 10.1111/cge.12986 28155232

[18] Kapadia H, Mues GD, D’souza R. Genes affecting tooth morphogenesis. Orthod Craniofacial Res. 2007;10(3):105–13. 10.1111/j.1601-6343.2007.00395.x 17651126

[19] Wacholder S, Chanock S, Garcia-Closas M, El Ghormli L, Rothman N. Assessing the probability that a positive report is false: an approach for molecular epidemiology studies. J Natl Cancer Inst. 2004;96(6):434–42. 10.1093/jnci/djh075 PMC7713993 15026468

[20] Wakefield J. A Bayesian measure of the probability of false discovery in genetic epidemiology studies. Am J Hum Genet. 2007;81(2):208–27. 10.1086/519024 PMC1950810 17668372

[21] Peres RCR, Scarel-Caminaga RM, Santo ARD, Line SRP. Association between PAX-9 promoter polymorphisms and hypodontia in humans. Arch Oral Biol. 2005;50(10):861–71. 10.1016/j.archoralbio.2005.02.003 16137495

[22] Liu H, Zhang J, Song S, Zhao H, Han D, Feng H. A case–control study of the association between tooth-development gene polymorphisms and non-syndromic hypodontia in the Chinese Han population. Eur J Oral Sci. 2012;120(5):378–85. 10.1111/j.1600-0722.2012.00986.x 22984994

[23] Jobbagy-Ovari G, Paska C, Stiedl P, Trimmel B, Hontvari D, Soos B, et al. Complex analysis of multiple single nucleotide polymorphisms as putative risk factors of tooth agenesis in the Hungarian population. Acta Odontol Scand. 2014;72(3):216–27. 10.3109/00016357.2013.822547 23964635

[24] Pan Y, Wang L, Ma J, Zhang W, Wang M, Zhong W, et al. PAX9 polymorphisms and susceptibility to sporadic tooth agenesis: a case–control study in southeast China. Eur J Oral Sci. 2008;116(2):98–103. 10.1111/j.1600-0722.2007.00517.x 18353002

[25] Isman E, Nergiz S, Acar H, Sari Z. PAX9 polymorphisms and susceptibility with sporadic tooth agenesis in Turkish populations: a case–control study. BMC Genomics. 2013;14:733. 10.1186/1471-2164-14-733 PMC3826669 24160254

[26] Abu-Siniyeh A, Khabour OF, Owais AI. The role of PAX9 promoter gene polymorphisms in causing hypodontia: a study in the Jordanian population. Appl Clin Genet. 2018;11:145–9. 10.2147/TACG.S183212 PMC6254497 30538524

[27] Al-Ani AH, Antoun JS, Thomson WM, Topless R, Merriman TR, Farella M. Common variants of EDA are associated with non-syndromic hypodontia. Orthod Craniofacial Res. 2021;24(1):155–63. 10.1111/ocr.12419 32772440

[28] Fernandez CCA, Pereira C, Ferreira F, Maciel JVB, Modesto A, Costa MC, et al. IRF6, MSX1, TGFA, dental anomalies, and skeletal malocclusion. Eur J Orthod. 2021;43(4):478–85. 10.1093/ejo/cjaa064 33200192

[29] Lancia M, Machado RA, Dionísio TJ, Garib DG, Santos CFD, Coletta RD, et al. Association between MSX1 rs12532 polymorphism with nonsyndromic unilateral complete cleft lip and palate and tooth agenesis. Arch Oral Biol. 2020;109:104556. 10.1016/j.archoralbio.2019.104556 31568994

[30] Mártha K, Kerekes Máthé B, Moldovan VG, Bănescu C. Study of rs12532, rs8670 polymorphism of Msh homeobox 1 (MSX1), rs61754301, rs4904155 polymorphism of paired box gene 9 (PAX9), and rs2240308 polymorphism of axis inhibitor protein 2 (AXIN2) genes in nonsyndromic hypodontia. Biomed Res Int. 2019;2019:2183720. 10.1155/2019/2183720 PMC6875315 31781599

[31] Wang H, Wang L, Pan Y-C, Ma J-Q, Zhang W-B. Msh homebox-1 polymorphisms and susceptibility to 198 sporadic tooth agenesis: a case–control study. Zhonghua kou Qiang yi xue za zhi = Zhonghua Kouqiang Yixue Zazhi = Chin J Stomatol. 2010;45(3):135–40. 20450679

[32] Brook AH. Multilevel complex interactions between genetic, epigenetic and environmental factors in the aetiology of anomalies of dental development. Arch Oral Biol. 2009;54(Suppl 1):S3–17. 10.1016/j.archoralbio.2009.09.005 PMC2981858 19913215

[33] Peters H, Balling R. Teeth. Where and how to make them. Trends Genet: TIG. 1999;15(2):59–65. 10.1016/s0168-9525(98)01662-x 10098408

[34] Zhang Z, Song Y, Zhao X, Zhang X, Fermin C, Chen Y. Rescue of cleft palate in Msx1-deficient mice by transgenic Bmp4 reveals a network of BMP and Shh signaling in the regulation of mammalian palatogenesis. Development (Cambridge, England). 2002;129(17):4135–46. 10.1242/dev.129.17.4135 12163415

[35] Zhang W, Qu HC, Zhang Y. Association of MSX1 and TGF-β1 genetic polymorphisms with hypodontia: meta-analysis. Genet Mol Res: GMR. 2014;13(4):10007–16. 10.4238/2014.November.28.5 25501212

[36] Zhang W, Qu HC, Zhang Y. PAX-9 polymorphism may be a risk factor for hypodontia: a meta-analysis. Genet Mol Res: GMR. 2014;13(4):9997–10006. 10.4238/2014.November.28.4 25501211

[37] Intarak N, Tongchairati K, Termteerapornpimol K, Chantarangsu S, Porntaveetus T. Tooth agenesis patterns and variants in PAX9: a systematic review. Jpn Dent Sci Rev. 2023;59:129–37. 10.1016/j.jdsr.2023.04.001 PMC10163602 37159578

[38] Neparáczki E, Juhász Z, Pamjav H, Fehér T, Csányi B, Zink A, et al. Genetic structure of the early Hungarian conquerors inferred from mtDNA haplotypes and Y-chromosome haplogroups in a small cemetery. Mol Genet Genomics. 2017;292(1):201–14. 10.1007/s00438-016-1267-z 27803981

[39] Chen Y, Bei M, Woo I, Satokata I, Maas R. Msx1 controls inductive signaling in mammalian tooth morphogenesis. Development (Cambridge, England). 1996;122(10):3035–44. 10.1242/dev.122.10.3035 8898217

